# Neutral Competition for *Drosophila* Follicle and Cyst Stem Cell Niches Requires Vesicle Trafficking Genes

**DOI:** 10.1534/genetics.117.201202

**Published:** 2017-05-16

**Authors:** Matthew S. Cook, Coralie Cazin, Marc Amoyel, Shinya Yamamoto, Erika Bach, Todd Nystul

**Affiliations:** * Center for Reproductive Sciences, University of California, San Francisco, California 94143-0452; † Department of Anatomy, University of California, San Francisco, California 94143-0452; ‡ Department of Obstetrics, Gynecology and Reproductive Sciences, University of California, San Francisco, California 94143-0452; § The Helen and Martin Kimmel Center for Stem Cell Biology, New York University School of Medicine, New York 10016; ** Department of Biochemistry and Molecular Pharmacology, New York University School of Medicine, New York 10016; †† School of Cellular and Molecular Medicine, University of Bristol, BS8 1TD, United Kingdom; ‡‡ Department of Molecular and Human Genetics, Program in Developmental Biology, Baylor College of Medicine, Houston, Texas 77030; §§ Jan and Dan Duncan Neurological Research Institute, Texas Children’s Hospital, Houston, Texas 77030

**Keywords:** cyst stem cell, follicle stem cell, neutral competition, vesicle trafficking

## Abstract

The process of selecting for cellular fitness through competition plays a critical role in both development and disease. The germarium, a structure at the tip of the ovariole of a *Drosophila* ovary, contains two follicle stem cells (FSCs) that undergo neutral competition for the stem cell niche. Using the FSCs as a model, we performed a genetic screen through a collection of 126 mutants in essential genes on the *X* chromosome to identify candidates that increase or decrease competition for the FSC niche. We identified ∼55 and 6% of the mutations screened as putative FSC hypo- or hyper-competitors, respectively. We found that a large majority of mutations in vesicle trafficking genes (11 out of the 13 in the collection of mutants) are candidate hypo-competition alleles, and we confirmed the hypo-competition phenotype for four of these alleles. We also show that *Sec16* and another COPII vesicle trafficking component, Sar1, are required for follicle cell differentiation. Lastly, we demonstrate that, although some components of vesicle trafficking are also required for neutral competition in the cyst stem cells of the testis, there are important tissue-specific differences. Our results demonstrate a critical role for vesicle trafficking in stem cell niche competition and differentiation, and we identify a number of putative candidates for further exploration.

## Abstract (precis)

Stem cell niche competition a common but poorly understood process. One impediment to understanding is a lack of useful niche competition alleles. In...

ADULT stem cells are defined by their ability to divide with asymmetric outcomes to self-renew and replenish differentiated cells that are lost from the tissue (Losick *et al.* 2011). This segregation of cell fates is achieved by a specialized microenvironment, or niche, that promotes the stem cell fate while allowing daughter cells to exit the niche and differentiate. However, stem cells can also be lost due to cell death or differentiation, and thus must be replaced by the daughter of a neighboring stem cell lineage to ensure that a healthy pool of stem cells is preserved throughout adulthood. In wild-type tissue, each stem cell has an equivalent chance of replacing its neighbor, resulting in a stochastic and unbiased pattern of replacement that can be described by a model of neutral competition for niche occupancy (Klein and Simons 2011). However, some mutations can confer a competitive advantage or disadvantage to a stem cell lineage relative to the neighboring wild-type stem cells, resulting in nonneutral, or biased, competition for niche occupancy (Vermeulen *et al.* 2013; Amoyel *et al.* 2014; Kronen *et al.* 2014; Snippert *et al.* 2014). The identification of these mutations demonstrates that competition for the stem cell niche is a genetically controlled process.

The follicle stem cells (FSCs) in the *Drosophila* ovary are a highly tractable model of stem cell niche competition (Losick *et al.* 2011; Sahai-Hernandez *et al.* 2012). The *Drosophila* ovary is comprised of long strands of developing follicles, called ovarioles, and a pair FSCs resides at the anterior tip of each ovariole in a structure called the germarium (Figure 1A) (Margolis and Spradling 1995; Nystul and Spradling 2007). These FSCs divide during adulthood to provide the follicle cells that surround germ cell cysts during follicle formation. FSCs are regularly lost and replaced during adulthood (Margolis and Spradling 1995), and several studies have identified genes that increase the rate of FSC loss. In most cases, the mutations investigated in these studies disrupt the ability of the mutant FSC to adhere to the niche or transduce niche signals and thus are presumed to cause the mutant stem cell to be lost in a cell-autonomous manner. However, the suggestion that stem cells may compete with the daughters of neighboring stem cells for niche occupancy raises the possibility that a mutation in a competing mutant lineage could act in a noncell-autonomous manner to influence the likelihood that a neighboring wild-type lineage will be lost and replaced.

**Figure 1:**
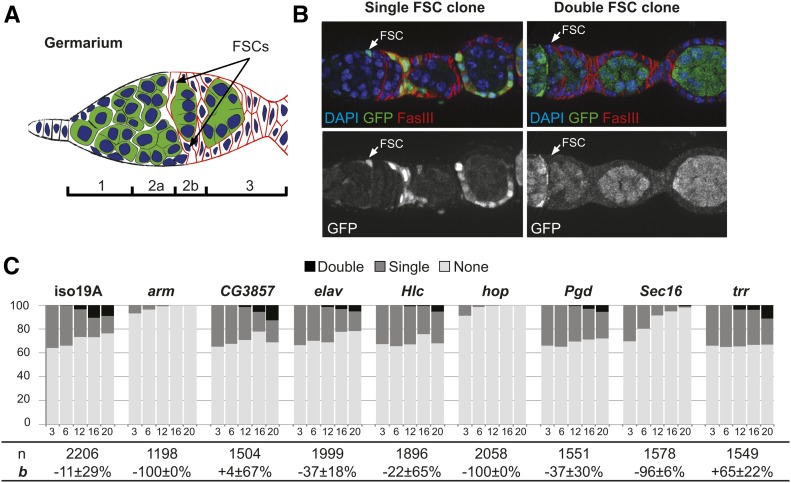
(A) Schematic of the germarium. The germarium is divided into four regions (1, 2a, 2b, and 3), and contains two FSCs at the region 2a/2b border. (B) Representative images of a mosaic germarium with one FSC marked by the lack of GFP (“single FSC clone”) and a fully marked germarium with both FSCs marked by the lack of GFP (“double FSC clone”). (C) Results from the pilot screen assayed at 3, 6, 12, 16, and 20 days ACI. *arm*, *hop*, and *sec16* have hypo-competition phenotypes.

To investigate this aspect of stem cell replacement further, we confirmed in a previous study (Kronen *et al.* 2014) that the pattern of FSC loss and replacement in wild-type tissue conforms to the neutral competition model, as expected (Margolis and Spradling 1995), and we expanded the model to include an additional parameter, *bias* (*b*), to quantify the effect of niche competition mutants. A bias of *b* = 0 indicates neutral competition, and the values of *b* range from −100 to +100%, with negative values indicating a “hypo-competition” phenotype (decreased fitness relative to wild type) and positive values indicating a “hyper-competition” phenotype (increased fitness relative to wild type). However, much remains unknown about the mechanism of niche competition and a limiting factor in the effort to understand this process has been a lack of hypo-competition mutations that do not directly disrupt niche adhesion or signaling, and a lack of hyper-competition mutations of any kind. Two previous screens for mutations that affect the rate of FSC loss and replacement either yielded only a small number of candidates (Kronen *et al.* 2014) or were not conducted in a manner that is compatible with analysis using the statistical model of niche competition (Wang *et al.* 2012).

Thus, to identify genes that regulate FSC niche competition, we conducted a screen through 126 recessive lethal mutations from a collection of alleles on the *X* chromosome (Haelterman *et al.* 2014; Yamamoto *et al.* 2014). This collection was generated by EMS mutagenesis of an isogenized line, and has been carefully curated to enrich for informative mutations. Specifically, each line in the collection was selected from a pool of >30,000 lines based on several criteria, including that the mutation does not cause cell lethality, that mutant clones produce a scorable phenotype in an adult tissue, and that the mutation mapped to a single gene and could be rescued with a defined genomic duplication. Here we identify one new hyper-competition allele, four new hypo-competition alleles, and many additional candidates that are likely to cause hypo- or hyper-competition. In addition, we show that alleles of genes involved in both exocytosis and endocytosis cause hypo-competition in the FSC niche; whereas neutral competition for the stem cell niche in the testis requires *Sec16*, a regulator of exocytosis, but not other the vesicle trafficking genes associated with competition for the FSC niche.

## Materials and Methods

### *X* chromosome mutant collection and screen matings

An isogenized y^1^ w* P{neoFRT}19A (*y w FRT19A*) control line and a collection of 126 mutant fly stocks as described in Haelterman *et al.* (2014) and Yamamoto *et al.* (2014) were acquired directly from Hugo Bellen’s laboratory as well as from the Bloomington *Drosophila* Stock Center. Male *w^122^ hsFlp Ubi-GFP FRT19A* flies were mated to y^1^ w* mut* P{neoFRT}19A/FM7c (where mut* represents the relevant mutation) virgin females. The offspring of these crosses were heat shocked and only females without *FM7c* were dissected.

### Fly husbandry

Fly stocks were maintained at either room temperature or 25° on standard molasses food. The following genotype was used for generating FSC clones in the primary screen and retesting: w^122^ hsFlp Ubi-GFP FRT19A/y^1^ w* mut* P{neoFRT}FRT19A. For clone generation in the testis, we used *y w hsflp*, *UAS-GFP*, *Tub > Gal4*; *Tub > Gal80 FRT40A* crossed to *FRT40A*; *UAS-RNAi* or *y w hsflp*, *UAS-GFP*, *Tub > Gal4*;; *Tub > Gal80*, *FRT2A* crossed to *UAS-RNAi*; *FRT2A*.

Stocks were obtained from the Bloomington Stock Center (BL), FlyORF, the Vienna *Drosophila* Resource Center (VDRC), or laboratories as indicated. The following stocks were used for Gal4 experiments in the ovary: (1) *Sec16 RNAi* (BL #53917), (2) *Sar1 RNAi* (BL #32364), (3) *UAS-Sar1* (FlyORF #F002032), (4) *Sec23 RNAi* (BL #32365), (5) *Sec31 RNAi* (BL #32878), (6) *UAS-Sec24-GFP* (from Stefan Luschnig), and (7) *UAS-Sec31-cherry* (from Stefan Luschnig). The following stocks were used for Gal4 experiments in the testis: (1) *Traffic jam-Gal4* (from Dorthea Godt), (2) *Crag RNAi* (BL #33594), (3) *Sec16 RNAi* (VDRC #109645), and *shi RNAi* (BL #28513).

### Pilot screen

We generated clones in seven cohorts of each genotype, and dissected ovaries from each cohort at two of five possible time points after clone induction (ACI): two cohorts at 3 and 12 days ACI, two cohorts at 6 and 12 days ACI, one cohort 12 and 16 days ACI, and two cohorts at 12 and 20 days ACI. We then dissected and stained with antibodies against GFP and FasIII to identify GFP^−^ clones and follicle cells, and used a statistical model of FSC competition to calculate the rate of FSC replacement and the bias (*b*) of the marked (GFP^−^) cells *vs.* the unmarked (GFP^+^) wild-type cells for niche occupancy.

### Clone induction

For ovarian clone induction, recently eclosed flies (0–5 days) from the appropriate matings were heat shocked for 1 hr in a 37° water bath twice daily (once in the morning, once in the afternoon) for 2 days (four heat shocks in total). Flies were provided fresh wet yeast after each heat shock event. For cyst stem cell (CySC) clone induction, freshly eclosed adult males were aged for 1 day and then heat shocked for 1 hr at 37°. Post induction, flies were maintained at 25° and given fresh wet yeast every other day as well as any day preceding dissection.

### Dissection, immunostaining, and imaging

Ovaries were dissected in 1× phosphate buffered saline (PBS) with CaCl_2_ and MgCl_2_ (CCFAL001-143502; University of California, San Francisco Cell Culture Facility). Ovarioles were teased apart at the anterior end using a 27 1/2 gauge needle tip (305109; Becton Dickinson) but left intact at the posterior end for easier processing. Ovaries for all experiments were then fixed in 4% paraformaldehyde (PFA) in 1× PBS for 15–90 min at room temperature (16% PFA stock from Electron Microscopy Sciences; 15710). Samples were then washed twice in 1× PBS plus 0.1% Tween (BP337-500; Fisher Scientific, Pittsburgh, PA) and then blocked for at least 1 hr at room temperature in 1× PBS plus 0.5% bovine serum albumin (BSA) (35% stock from Sigma Chemical, St. Louis, MO; A7979-50ML) and 0.1% Tween. Samples were incubated in primary antibody diluted in 500 μl 1× PBS plus 0.1% BSA and 0.1% Tween (1× PBS-BT) overnight at 4° on a rotating platform. The next day samples were rinsed once in 500 μl 1× PBS-BT, washed three times for at least 10 min each in 500 μl 1× PBS-BT, and incubated with secondary antibody diluted in 500 μl 1× PBS-BT overnight at 4° or ∼2 hr at room temperature. Samples were then washed four times for at least 10 min each in 1× PBST-BT and suspended in a drop of Vectashield with DAPI (H-1200; Vector Laboratories, Burlingame, CA). The samples were then mounted on glass slides immediately or stored at 4° for up to 2 months prior to mounting.

Testes dissections and immunohistochemistry were performed as previously described (Flaherty *et al.* 2010). CySCs were scored as Zfh1- or Traffic jam (Tj)-positive cells one cell diameter away from the hub. At least 20 testes of each genotype were scored, and in most experiments there were 40–60 testes per genotype scored. For lineage-wide depletion, control or Tj > Sec16 RNA interference (RNAi) males were upshifted to 29° for 10 days 1 day after eclosion.

The following primary antibodies and dilutions were used: guinea pig anti-GFP (132005, 1:1000) from Synaptic Systems; mouse anti-Dlg (4F3, 1:200), mouse anti-FasIII (7G10, 1:200), and rat anti-DE-cadherin (DCAD2, 1:100) from the Developmental Studies Hybridoma Bank; rabbit anti-aPKC (SC-216, 1:200), rabbit anti-Vasa (SC-30210, 1:1000), and goat anti-Vasa (SC-26877, 1:100) from Santa Cruz Biotechnologies; Rabbit anti-pERK (4370, 1:100) from Cell Signaling Technologies; rabbit anti-Sec16 (gift from Catherine Rabouille’s laboratory, 1:1000); rabbit anti-GFP (TP401, 1:1000) from Torrey Pines Biolabs; mouse anti-GFP (A-11120, 1:500) from Thermo Fisher Scientific; and guinea pig anti-Tj (1:3000, gift of Dorothea Godt). The secondary antibodies were from Invitrogen (Carlsbad, CA) (for ovaries) or Jackson ImmunoResearch (testes).

All ovary samples were imaged on a Carl Zeiss (Thornwood, NY) upright compound light microscope with Apotome using a halogen lamp (Lumen Dynamics, X-Cite 120PC Q) and analyzed with Axiovision 4.8 software. Testes samples were scanned on a Carl Zeiss LSM510 confocal at 63×.

### Statistics

The bias values, 95% confidence intervals, and associated *P*-values were calculated as described previously (Kronen *et al.* 2014) by inputting the number of germaria with 0, 1, or 2 marked FSCs at each time point and the number of days ACI of each time point into a MatLab script (provided in Supplemental Material, File S1). For analysis of each line at the single time point (13 days ACI) used in the screen, the number of marked and unmarked FSCs observed in each line was compared to the isogenized control from the same round using a Pearson’s Chi-squared test in R.

### Data availability

Strains and reagents are available upon request. MatLab scripts for calculations of bias and *P*-values are provided as File S1.

## Results

### Pilot screen establishes method for high throughput screen and identifies new hypo-competition alleles

To identify mutations that cause hypo- or hyper-competition for the FSC niche, we first conducted a pilot screen using eight candidate alleles and an isogenized wild-type control. For each allele, we performed a standard FSC niche competition assay in which sparse FSC clones are generated in adult flies using a heat shock-flippase/FRT system, and the number of germaria with 0 (none), 1, or 2 (both) marked FSCs are quantified at multiple time points ACI. FRT recombination produces wild-type or homozygous mutant clones that are marked, in this case, by the lack of GFP expression (GFP^−^), and FSC clones can be identified as large contiguous groups of marked cells that extend from the region 2a/2b border through the germarium and into the budded follicles (Figure 1B). In germaria where one FSC is marked initially, the replacement of one FSC with a daughter of the other FSC leads to an outcome in which neither or both FSCs are marked. Thus, the change in the frequency of germaria with one marked FSC can be used to calculate the rate of turnover.

We generated clones in seven cohorts of each genotype and quantified the FSC clone frequencies at 3, 6, 12, 16, and 20 days ACI. To reduce the potential effect of variation in clone induction frequency between cohorts, we dissected ovaries from each cohort at two of the five time points in an overlapping manner (see *Materials and Methods*). We then stained with antibodies against GFP and FasIII to identify GFP^−^ clones and follicle cells, and used a statistical model of FSC competition to calculate the bias (*b*) of the marked (GFP^−^) cells *vs.* the unmarked (GFP^+^) cells for niche occupancy.

**Figure 2:**
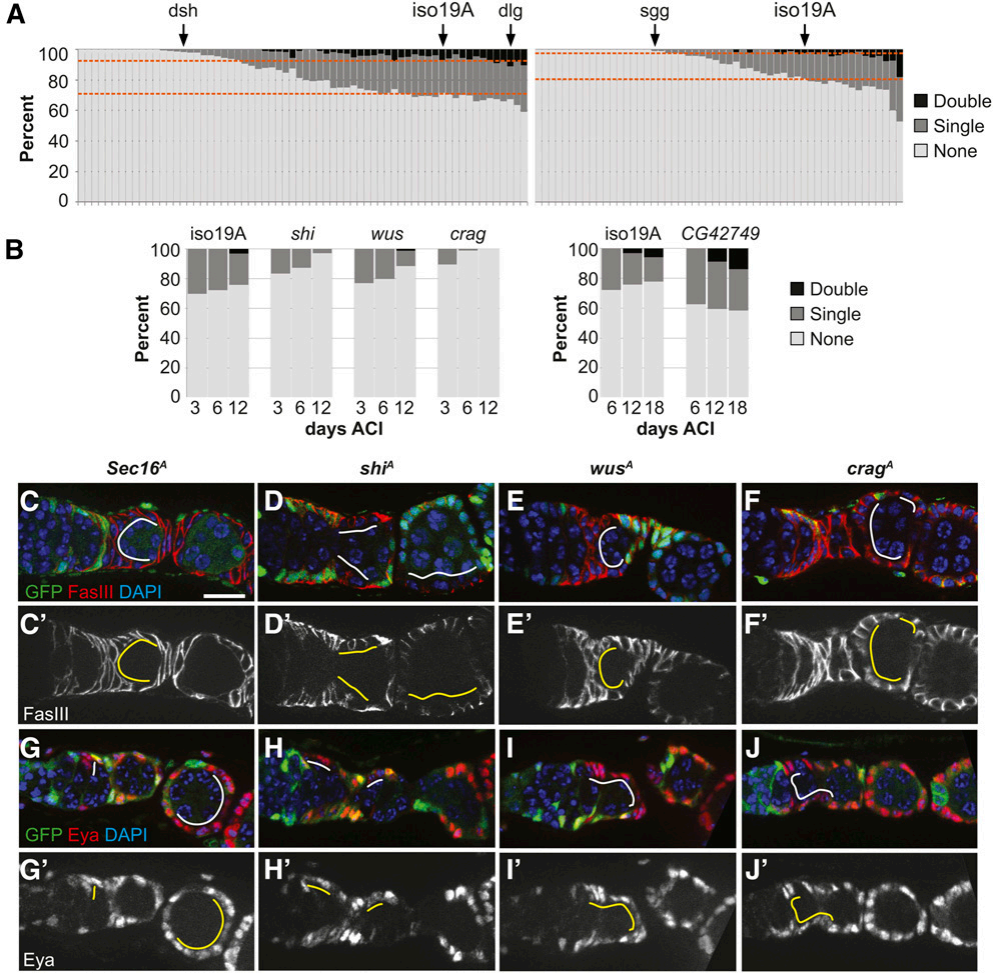
(A) Screen results. Dashed lines represent the “double”/“single” border and “single”/“none” border for the *isoFRT19A* control in each set. (B) Niche competition assays for four candidate alleles identified in the first part of the screen. Consistent with the predictions from the primary screen, the *Crag*, *shi*, and *wus* alleles cause a decrease in FSC clone frequency, and the *CG42749* allele causes an increase in clone frequency. (C–J and C′-J′) GFP^−^ *Sec16^A^*, *shi^A^*, *wus^A^*, and *Crag^A^* FSC clones 6 days ACI stained for follicle cell markers (C–F) FasIII or (G–J) Eya (red), GFP (clonal marker, green), and DAPI (blue). Position of mutant follicle cells indicated by solid white and yellow lines. Mutant clones grow, form a single layered epithelium around the germ cell cysts, and express FasIII and Eya. Bar, ∼10 μm.

In the isogenized control, the percentage of germaria with 0 or 2 marked FSCs increased at similar rates (Figure 1C), indicating that control stem cells were equally likely to be lost from the niche and replaced by their neighbors as they were to replace the unlabeled stem cells. This result is consistent with neutral competition for niche occupancy between the marked and unmarked populations in wild-type tissue. Statistical analysis of the data using the neutral competition model confirmed the lack of a significant competition phenotype (Table 1; P = 0.21 for the null hypothesis that *b* = 0). Likewise, we found no significant deviation from neutral competition in germaria with FSC clones that were mutant for *CG3857*, *embryonic lethal abnormal vision* (*elav*), *Helicase* (*Hlc*), *Phosphogluconate dehydrogenase* (*Pgd*), or *trithorax related* (*trr*) (Table 1). In contrast, homozygosity for the mutant alleles of either *armadillo* (*arm*) or *hopscotch* (*hop*) caused maximal hypo-competition (*b* = −100%, *P* < 0.01; Figure 1C; Table 1). For these alleles, clone frequency rapidly declined to <1% of germaria with a clone at 12 days ACI and 0% at 16 and 20 days ACI. This is consistent with previous findings that these genes are required for FSC maintenance (Song and Xie 2003; Vied *et al.* 2012). We also found that FSC clones that were homozygous for *Sec16^A^*, an important regulator of COPII vesicle trafficking from the endoplasmic reticulum to the Golgi (Ivan *et al.* 2008), were strongly hypo-competitive (*b* = −97%, *P* = 0; Figure 1C; Table 1). Unlike with *arm^A^* and *hop^A^*, homozygous mutant *Sec16^A^* FSC clones were present at all time points tested. *Sec16^A^* mutant cells also expressed the follicle cell markers, FasIII and Eyes absent (Eya) (Figure 2C), and mutant FSC clones commonly extended across multiple follicles. These observations indicate that *Sec16^A^* is not cell lethal and, although it causes a strong hypo-competition phenotype, it does not completely disrupt FSC self-renewal, proliferation, or follicle cell differentiation.

**Table 1:** Bias values of lines tested in the pilot screen

Gene	Bias [95% C.I.]	*P*-value
19A control	−27% [0%, −45%]	0.215
*arm*	−100% [−100%, −61%]	0.001
*CG3857*	+28% [−16%, +92%]	0.515
*elav*	−43% [−60%, −14%]	0.102
*Hlc*	8% [−37%, +91%]	0.892
*hop*	−100% [−100%, −56%]	0.008
*Pgd*	−53% [−74%, +18%]	0.268
*Sec16*	−97% [−99%, −89%]	<10^−10^
*Trr*	+55% [+7, 96%]	0.149

### The screen identified new candidate hypo-competition and hyper-competition alleles

Multiple time points must be scored to determine whether a mutation causes a hypo- or hyper-competition phenotype, but a complete FSC niche competition assay is impractical for a large number of lines. However, by 12 days ACI, there were clearly fewer marked FSCs in the three lines with hypo-competition alleles compared to the isogenized control. This time point was assayed in seven independent trials in the pilot screen, and we found that a Chi-squared test for the significance of these differences at *P* < 0.01 was a strong predictor of whether the allele caused a significant competition phenotype (Table 2). Thus, we chose to use a single time point at a similar time (13 days ACI) for screening the remaining 118 mutant lines (Figure 2A). To maximize for consistency of clone induction and scoring, we divided the collection into two large sets and carried all the lines within each set through the procedure together. We included the isogenized control in each round, and an allele of *discs large 1* (*dlg1^m52^*) in round 1 as a test of the assay on a hyper-competition allele (Kronen *et al.* 2014). In addition, mutant alleles of genes required for FSC maintenance, such as *dishevelled^A^* (*dsh^A^*) in round 1 and *shaggy^A^* (*sgg^A^*) in round 2 (Figure 2A, arrows) provided a test of the assay on putative hypo-competition alleles (Song and Xie 2003). As expected, the frequencies of *dsh^A^* and *sgg^A^* FSC clones were significantly lower than the isogenized control (*P* < 0.01; Tables S1 and S2 in File S2), indicating that our assay was able to identify these putative hypo-competition alleles. The frequency of *dlg1^m52^* FSC clones was higher than in the isogenized control, as expected, and was in fact the third highest of all 67 lines scored in round 1 (Table S1 in File S2). The comparison to the isogenized control did not reach the same level of significance as the hypo-competition alleles in our pilot screen (*P* < 0.08 *vs.* *P* < 0.01), probably because hyper-competition generally has a smaller effect on FSC clone frequency than hypo-competition, so more clones would need to be scored to achieve the same level of statistical significance.

**Table 2:** *P*-values from a Chi-squared test of differences in the number of marked (GFP^−^) FSCs in the mutant *vs.* the paired control in each trial at 12 days ACI in the pilot screen

	Trial 1	Trial 2	Trial 3	Trial 4	Trial 5	Trial 6	Trial 7
*arm*	<10^−4^	<10^−10^	<10^−4^	<10^−3^	<10^−5^	<10^−10^	<10^−4^
*CG3857*	0.976	0.078	0.205	0.205	0.245	0.066	0.250
*elav*	0.483	0.086	0.290	0.474	0.009	0.845	0.007
*Hlc*	0.018	0.284	0.013	0.453	0.105	0.475	0.075
*hop*	<10^−11^	<10^−16^	<10^−13^	<10^−4^	<10^−14^	<10^−13^	<10^−4^
*Pgd*	0.445	0.039	0.138	0.275	0.129	0.554	0.684
*Sec16*	<10^−5^	<10^−7^	<10^−3^	<10^−2^	<10^−7^	<10^−7^	0.011
*Trr*	<10^−5^	0.916	0.085	<10^−4^	0.331	0.156	0.796

Gray shading indicates a *P*-value of <0.01. The three hypo-competitive alleles, in *Sec16*, *hop*, and *arm*, have *P*-values below this threshold in every trial, except trial 7 for Sec16. In contrast, alleles that do not cause hypo-competition rarely have *P*-values below this threshold.

We found that 65 lines had a significantly lower frequency of FSC clones (*P* < 0.01; Tables S1 and S2 in File S2) and thus contained candidate hypo-competition alleles. We also found seven lines (Table S1 and Table S2) with a higher frequency of FSC clones and *P*-values below that of *dlg1^m52^* (three of which had *P*-values <0.01) and thus are potential hyper-competition alleles. Since we found in the pilot screen that *Sec16^A^* causes hypo-competition, we were interested in other genes involved in vesicle trafficking. The collection of mutants contains alleles of 12 other vesicle trafficking genes: *adaptor protein-1 ɣ subunit* (*AP-1ɣ*) (Burgess *et al.* 2011), *β’COP*, *comatose* (*comt*) (Sanyal *et al.* 1999), *Calmodulin-binding protein related to a Rab3 GDP/GTP exchange protein* (*Crag*) (Denef *et al.* 2008), *deep orange* (*dor*) (Sevrioukov *et al.* 1999), *Rab connectin-3A* (*Rbcn-3A*), *Rbcn-3B* (Yan *et al.* 2009), *shibire* (*shi*) (Koenig and Ikeda 1996), *tempura* (*temp*) (Charng *et al.* 2014), *Vps26* (Seaman *et al.* 1998), *wurst* (*wus*) (Behr *et al.* 2007), and *Ykt6* (Tai *et al.* 2004) (Tables S1 and S2 in File S2, orange highlight). We found that 10 of these alleles are candidate hypo-competition mutations with significantly lower clone frequencies than the isogenized control (*P* < 0.01; Tables S1 and S2 in File S2).

### Candidate alleles were confirmed with full niche competition assays

To confirm a subset of the putative hits from round 1 of the screen, we performed niche competition assays with multiple time points on three candidate hypo-competition mutations of genes involved in vesicle trafficking (*Crag^A^*, *shi^A^*, and *wus^A^*) and one hyper-competition mutation, *CG42749^A^*, which is in an uncharacterized gene coding for a protein with a transmembrane domain and carbohydrate-binding module (Figure 2B). We found that the frequency of *Crag^A^*, *shi^A^*, and *wus^A^* FSC clones declined over a 12-day time course, and there were very few or no germaria with two marked FSCs at all time points. Analysis of the changes in clone frequencies indicated that these mutations have a strong negative bias (*b* = −100, −100, and −85%, respectively; *P* < 0.05; Table 3). The mutant FSC clones that were present typically grew to cover one or more follicles, indicating that these mutations are not cell lethal in the FSC lineage. Mutant follicle cells also expressed the follicle cell markers, FasIII and Eya (Figure 2, C–J). FasIII localized to the membrane in all four mutant genotypes (Figure 2, C–F), suggesting that these mutations do not completely abrogate vesicle trafficking, though FasIII localization was clearly disrupted in *shi^A^* follicle cells (Figure 2D). With the *CG42749^A^* line, the percentage of germaria with at least one marked FSC remained approximately constant over an 18-day time course, and the percentage of germaria with two marked FSCs increased substantially. Analysis of the changes in clone frequency indicated that this mutation is strongly hyper-competitive (*b* = +100, *P* < 0.05; Table 3). Therefore, the retest validated all four candidates, indicating that the single time point assay used in the screen is an accurate predictor of niche competition phenotypes.

**Table 3:** Bias values of vesicle trafficking alleles tested in the screen

Gene	Bias [95% C.I.]	*P*-value
19A control	+2% [+88%, −49%]	0.970
*Crag*	−100% [−67%, −100%]	0.000
*shibire*	−100% [−39%, −100%]	0.004
*wurst*	−85% [−39%, −95%]	0.024
*CG42749*	+100% [+30%, +100%]	0.012

### Vesicle trafficking genes are required for FSC self-renewal and follicle cell differentiation

Sec16 is a GTPase binding protein that colocalizes with COPII components at endoplasmic reticulum exit sites (ERESs) to catalyze vesicle formation (Ivan *et al.* 2008; Yorimitsu and Sato 2012). The *Sec16^A^* allele identified in our screen contains a single missense mutation (P1926S) in the conserved C-terminal domain of the protein. Although the conserved C-terminal domain is not thought to be required for its localization to ERESs (Yorimitsu and Sato 2012), homozygosity for *Sec16^A^* is lethal for the organism, indicating that it encodes for a dysfunctional protein. To further investigate how this mutation may affect Sec16 function, we first examined the localization of Sec16 in wild-type and mutant germaria.

**Figure 3:**
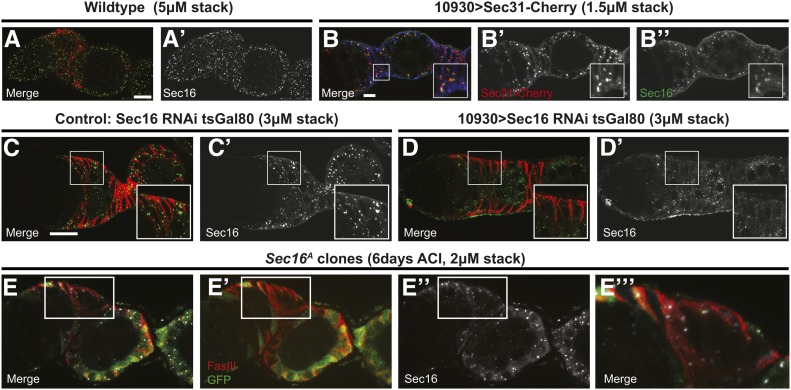
(A-B) Wildtype germaria stained for Sec16 (green) and FasIII (red). Sec16 localizes to discrete puncta throughout all cells in the germarium. (A′-D′) Sec16 channel only. (B) Overexpression of Sec31-Cherry (red) in follicle cells with 10930-Gal4 at 7 days post eclosion. Sec16 (green) colocalizes with Sec31 within the FasIII-positive (blue) follicle cells, indicating that it is at ERESs. Boxed region magnified in inset to show co-localization. (C-D) Germaria with UAS-sec16 RNAi and tub-Gal80^ts^ that lack a Gal4 driver as a control or have a 10930-Gal4 stained for Sec16 (green) and FasIII (red) 4 days after shift to 29°. Sec16 puncta are visible in the control (C) but are absent specifically in the follicle cells (as indicated by FasIII, red) of germaria expressing Sec16 RNAi (D). Boxed regions magnified in insets to show loss of Sec16 puncta in upon RNAi knockdown. (E) Germarium with a GFP^−^ *sec16^A^* FSC clone stained for GFP (the clonal marker, green), FasIII (red), and Sec16 (white). Puncta of Sec16 are clearly visible in *sec16^A^* (GFP^−^) follicle cells. White box outlines a region with GFP^−^ follicle cells and is magnified in E′′′. (E′) FasIII and GFP channels, (E′′) Sec 16 channel. White scale bar is approximately 10 μm.

**Figure 4:**
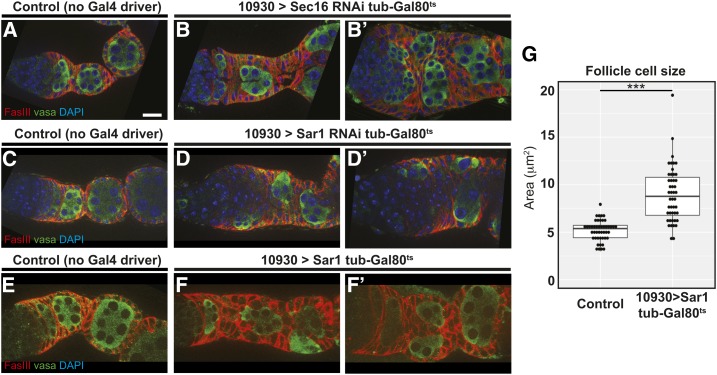
(A–F) Germaria with tub-Gal80^ts^ and UAS-sec16 RNAi, UAS-sar1 RNAi, or UAS-sar1 that lack a Gal4 driver as a control (A, C, and E) or have 10930-Gal4 (B-B′, D-D′, and F-F′) stained for FasIII (red) and Vasa (green) 7 days after shift to 29°. Knockdown of either *Sec16* or *sar1* or overexpression of *sar1* causes severe morphological defects in the follicle epithelium that leads to follicle cell multilayering and cyst encapsulation defects. (G) Quantification of follicle cell sizes in germaria from the populations represented in (E and F). Cell sizes were calculated by tracing the cell boundary visible in a single optical section of six follicle cells in each germarium and measuring the area within. Each ● represents the average of the areas of the six cells from each germarium. *N* = 40–50 germaria taken from two independent preparations of each genotype. *** *P* < 0.001. Bar, ∼10 μm.

In wild-type germaria, Sec16 localized to discrete cytoplasmic puncta that were noticeably brighter and larger in the follicle cells (Figure 3A). Expression of *Sec16^RNAi^* with a follicle cell driver, 10930-Gal4 (Hartman *et al.* 2010), eliminated these puncta in follicle cells, indicating that the antibody is specific and that RNAi knockdown substantially reduces the protein levels (Figure 3, C and D). In addition, we found that these puncta colocalized with ectopically expressed Sec31::mCherry (Figure 3B) (Forster *et al.* 2010), verifying that they are ERESs. Interestingly, we found that Sec16 expression and localization appeared normal in *Sec16^A^* FSC clones (Figure 3E). This is consistent with the previous observation that the C-terminal domain is not required for localization, and indicates that ERESs can still form in these mutant cells (Yorimitsu and Sato 2012). In contrast, knockdown of *Sec16* expression by RNAi specifically in adult follicle cells produced severe phenotypes, including an accumulation and multilayering of follicle cells, and defects in the formation and budding of follicles from the germarium (Figure 4, A and B). Sec16 promotes vesicle formation by preventing Sec23 and Sec31 from activating the GTPase activity of Sar1 (Lord *et al.* 2013). Expression of *Sec23* or *Sec31* RNAi (Ni *et al.* 2011) with 10930-Gal4 did not produce a phenotype in the follicle epithelium, though we did not have antibodies against these proteins to assess the knockdown efficiency. In addition, overexpression of Sec31 or another COPII component, Sec24, also did not produce a phenotype. However, both RNAi knockdown and overexpression of Sar1 produced follicle budding phenotypes, (Figure 4, C–F) and Sar1 overexpression also significantly increased follicle cell size compared to wild-type controls (Figure 4G). We attempted to generate clones for a niche competition assay using a null allele, *Sar1^11-3-63^* (Massarwa *et al.* 2009), but were unable to recover any GFP^−^ FSC clones, suggesting that this mutation is either cell lethal or severely impairs clone growth and retention. Taken together, these observations indicate that proper regulation of vesicle trafficking is essential for FSC niche competition and follicle cell differentiation. Moreover, the comparison between the mild phenotypes we observed in *Sec16^A^* follicle cell clones and the more severe phenotypes produced by Sec16 RNAi indicate that *Sec16^A^* is a hypomorphic allele.

**Figure 5:**
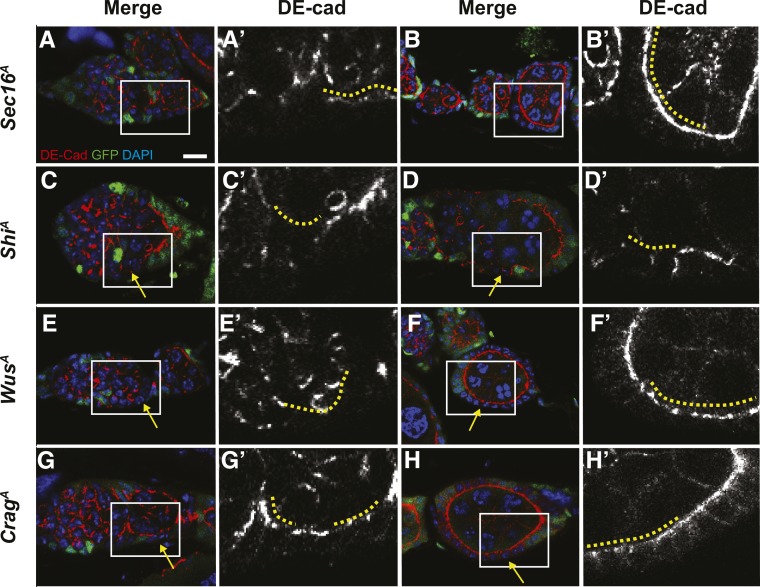
Germaria with *Shi^A^* (A-B), *Wus^A^* (C-D), *Crag^A^* (E-F), or *Sec16^A^* (G-H) clones stained for GFP (clonal marker, green), DE-cad (red), and DAPI (blue). DE-cad channel shown separately in A′-H′. Images of clones in the germarium shown in A, C, E, and G; images of clones in budded follicles shown in B, D, F, and H. The positions of mutant follicle cells are indicated by solid yellow lines. Boxed regions in A-H are magnified in A′-H′. Yellow arrows indicate GFP^−^ homozygous mutant follicle cells. DE-cad is present on the apical membranes of heterozygous, GFP^+^ follicle cells in all cases but absent from the apical domains of *Shi^A^* follicle cells in the germarium and in budded follicles; absent from the apical domains of *Wus^A^* follicle cells in the germarium and disrupted in *Wus^A^* follicle cells of budded follicles; present but mildly disrupted in *Crag^A^* and *Sec16^A^* follicle cells in the germarium; and normally localized in *Crag^A^* and *Sec16^A^* follicle cells in budded follicles.

To further investigate the follicle cell phenotype of *Sec16^A^* and to determine whether homozygosity for *shi^A^*, *wus^A^*, or *Crag^A^* also causes phenotypes associated with vesicle trafficking, we assayed for the localization of a membrane protein, DE-cadherin (DE-cad), to the plasma membrane. DE-cad localization to the plasma membrane is dependent on vesicle trafficking (Woichansky *et al.* 2016) and is clearly detectable on the apicolateral membranes of follicle cells starting in region 3 of the germarium (Franz and Riechmann 2010; Kronen *et al.* 2014). In mosaic germaria, GFP^+^ cells that are heterozygous for the mutation are intermingled with GFP^−^ homozygous mutant cells, so both genotypes can be assayed in the same tissue. We found that DE-cad was properly localized to the apicolateral membrane of heterozygous (GFP^+^) follicle cells in all cases, but was disrupted in the homozygous mutant (GFP^−^) cells to different extents in each of the vesicle trafficking mutant lines at 6 days ACI (Figure 5). Homozygosity for *shi^A^* produced the strongest phenotype, with a complete absence of apicolateral DE-cad in follicle cells in both the germarium and in budded follicles (Figure 5, A and B). In *wus^A^* clones, apicolateral DE-cad was absent in follicle cells in the germarium and present, but less uniform, in the follicle cells of budded follicles (Figure 5, C and D); whereas in *Crag^A^* and *Sec16^A^* clones, we observed a mild disruption of apicolateral DE-cad in follicle cells in the germarium but no effect in budded follicles (Figure 5, E–H). These observations suggest that vesicle trafficking is impaired by all four of these alleles, though to a greater extent by *shi^A^* and *wus^A^* than by *Crag^A^* and *Sec16^A^*.

### Hypo-competition alleles of vesicle trafficking genes disrupt niche signaling

**Figure 6:**
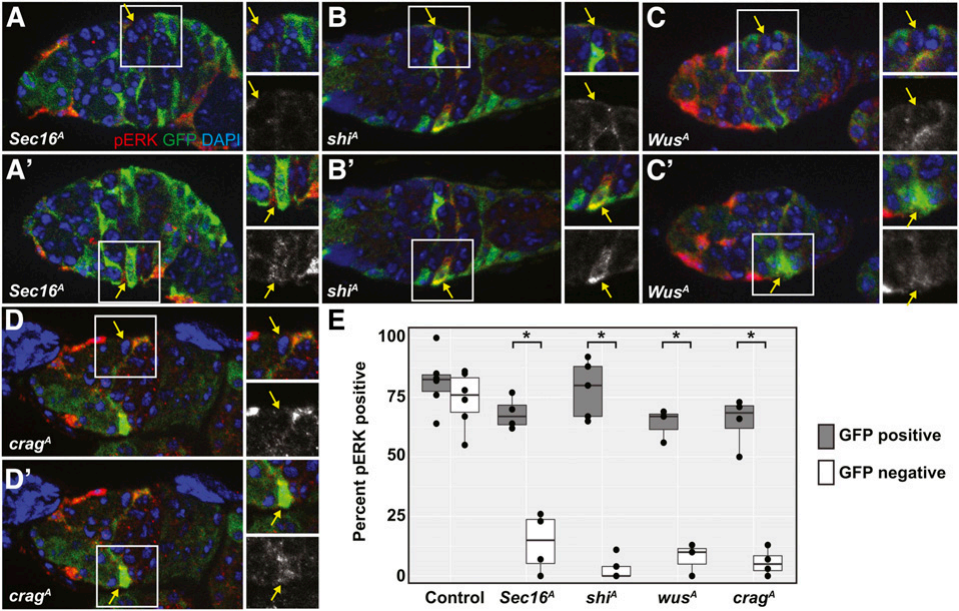
(A–D) Germaria with (A) *Sec16^A^*, (B) *Shi^A^*, (C) *Wus^A^*, or (D) *Crag^A^* clones stained for pERK (red), GFP (clonal marker, green), and DAPI (blue). Images are oriented so that the GFP^−^ FSC is at the top of the image as shown in (A–D), and the GFP^+^ FSC is at the bottom as shown in (A′–D′). Each FSC (indicated by a yellow arrow within the white boxes) is shown in the insets to the right of each image. The insets show a merge of all three channels, and the pERK channel alone. Each image is an optical section selected from a *z*-stack to best capture the indicated FSC. For all four genotypes, the homozygous mutant GFP^−^ FSC has low or undetectable pERK staining, and the heterozygous GFP^+^ FSC in the same germarium has normal levels of pERK staining. (E) Quantification of the frequency of pERK staining in FSCs that are heterozygous (GFP^+^, gray boxes) or homozygous (GFP^−^, white boxes) for the chromosome arm with the allele indicated on the *x*-axis. Three to five independent trials were conducted for each genotype; each ● indicates the mean values from a single trial. *N* = 53–77 germaria scored (total from all three to five trials) for each genotype. Boxes show the mean and the first and third quartiles of the means from each trial. * *P* < 0.01.

We found previously that EGFR signaling is a necessary and specific FSC niche signal. Phosphorylated ERK (pERK), an indicator of EGFR pathway activity, is clearly detectable in FSCs but not in prefollicle cells that have moved downstream from the niche, and *Egfr* is essential for FSC maintenance in the niche but not for early prefollicle cell differentiation (Castanieto *et al.* 2014). To determine whether the hypo-competition alleles of vesicle trafficking genes that we identified affect EGFR signaling in FSCs, we stained germaria with GFP^−^ wild-type or homozygous mutant FSC clones at 6 days ACI for pERK and GFP. We then compared the pERK staining of the marked (GFP^−^) and unmarked (GFP^+^) FSCs within the same tissue. As expected, we found that pERK was clearly detectable in the large majority of both GFP^−^ and GFP^+^ FSCs in the isogenized FRT19A control line (Figure 6E). In contrast, for the *Sec16^A^*, *shi^A^*, *Crag^A^*, and *wus^A^* lines, significantly fewer homozygous mutant (GFP^−^) FSCs were pERK^+^ compared to the heterozygous (GFP^+^) FSCs in the same tissues (Figure 6). Thus, homozygosity for these hypo-competition alleles is associated with a decrease in EGFR signaling within FSCs.

### Knockdown of *Sec16*, *shi*, and *Crag* has distinct effects on competition, survival, and differentiation in cyst stem cells in the testis

Vesicle trafficking is required in many tissues for a wide variety of functions, though the type of trafficking and the specific genes involved can vary (Wirtz-Peitz and Zallen 2009; Tepass 2012). To determine whether some of the vesicle trafficking genes we identified are also important for neutral competition in another tissue, we assayed for phenotypes in CySCs in the *Drosophila* testis (Greenspan *et al.* 2015). The *Drosophila* testis is a blind-ended tube that contains all stages of spermatogenesis (Fuller 1998). The niche is comprised of quiescent somatic cells called hub cells, which are anchored to the apical tip of the testis. The niche supports ∼13 CySCs and 9–13 germline stem cells (GSCs) through the production of short-range self-renewal signals. CySCs are the only mitotically active somatic cells in the testis and express the transcription factors Zfh1 and Tj (Hardy *et al.* 1979; Li *et al.* 2003; Leatherman and Dinardo 2008). As CySC daughters differentiate, they exit the cell cycle and ensheath the daughter of a GSC, which can only differentiate upon proper somatic encystment (Sarkar *et al.* 2007; Lenhart and DiNardo 2015). As somatic cells differentiate, they move out of the niche as part of a germline cyst, lose expression of Zfh1 and Tj, and gain expression of Eya (Fabrizio *et al.* 2003). Like FSCs, CySCs compete with each other for niche occupancy (Issigonis *et al.* 2009; Singh *et al.* 2010; Amoyel *et al.* 2014).

**Figure 7:**
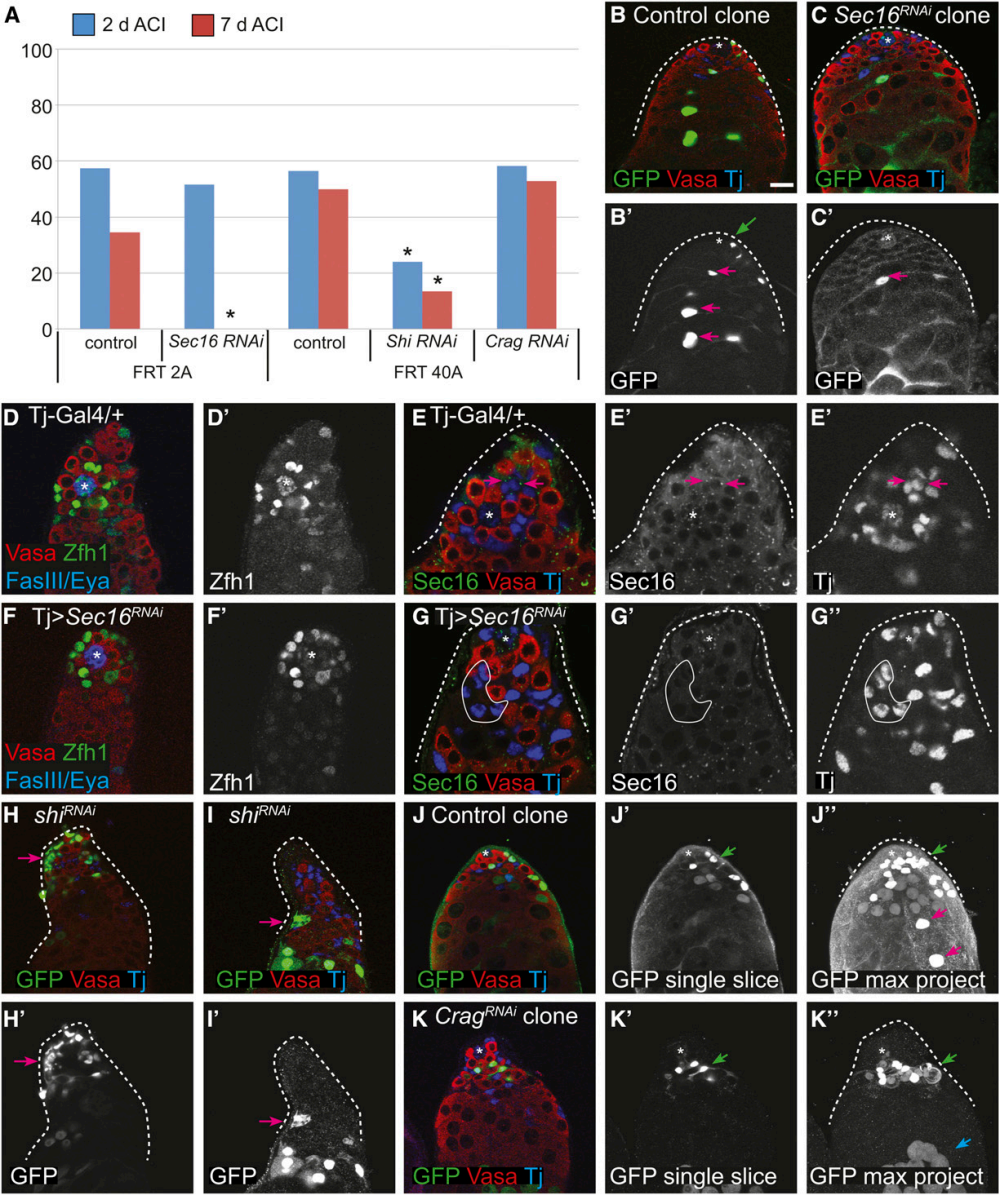
(A) CySC niche competition assays for control and RNAi knockdown of the indicated genes and matched *FRT* control clones at 2 (blue bars) and 7 (red bars) days ACI. * *P* < 0.05 compared to the control in the same set. (B and C) Control clones with CySCs [(B′), green ←] and differentiating daughter cells [(B′), pink ←] were recovered at 7 days ACI; whereas *Sec16* RNAi CySCs were not recovered at 7 days ACI, but their differentiating daughter cells were observed [(C′), pink ←]. (D–G) Depletion of Sec16 from the entire somatic lineage does not perturb the stem cell niche as a Tj > Sec16 RNAi testis (F and G) looks very similar to a control testis (D and E) in terms of the number of CySCs (Zfh1-positive cells). (D′ and F′) Zfh1 channel only. (G′-G′′) Sec16 and Tj channels only. Sec16 localizes to discrete cytoplasmic puncta in cyst cells of wild-type testes (E–E′), and this signal is eliminated in a Tj > Sec16 RNAi testis [(G), representative cyst cells outlined with a white line]. (H and I) *shi* RNAi CySC clones have abnormal phenotypes, including pronounced fragmentation that suggests the cells are dying [(H′), ←] and aberrant membrane formations that suggest incomplete germ cell ensheathment [(I′), ←]. (J and K) In a testis with control clones at 7 days ACI, a labeled CySC [(J′′), green ←] and labeled differentiating cyst cells [(J), pink ←] were readily observed. In contrast, in Crag RNAi clones, labeled CySCs were readily observed [(K′′), green ←] but differentiating Crag-depleted cyst cells were not seen. Note that the GFP cells at the bottom are labeled germ cells [(K′′), blue ←]. (J, J′, K, K′) are single confocal sections whereas (K′′) and (J′′) are maximum projections.

Since we could not perform clonal analysis of *X*-linked recessive lethal mutations in males, we used clonal or lineage-wide RNAi knockdown of Sec16, shi, or Crag instead. We confined our analyses to somatic cells, either CySCs clones or somatic lineage knockdown cells. We found that the frequency of testes with control CySC clones, marked by the expression of GFP, decreased slightly during the 7 days ACI; consistent with neutral competition for the stem cell niche in this tissue (Figure 7A). In contrast, CySC clones expressing Sec16 RNAi were rapidly lost, decreasing from 51.6% at 2 days ACI to 0% at 7 days ACI (Figure 7, A–C). The decrease in clone frequency could be because Sec16 is required for self-renewal or CySC competitiveness. To distinguish between these possibilities, we depleted Sec16 from the somatic lineage using the somatic-specific driver Tj-Gal4. We reasoned that if Sec16 was required for self-renewal, then its somatic depletion would cause loss of the somatic lineage. The RNAi knockdown was effective, as evidenced by a lack of Sec16 staining in the soma, but the tissue morphology was normal and we observed a full complement of Zfh1+ cells (Figure 7, D–F). Therefore, we conclude that knockdown of Sec16 in CySCs does not abrogate self-renewal but causes hypo-competition for the niche.

Likewise, CySC clones expressing shi RNAi were also rapidly lost from the niche, and there were significantly fewer clones at 7 days post heat shock than in the control group (Figure 7A). However, unlike with Sec16 RNAi, expression of shi RNAi clones caused multiple phenotypes, including cellular fragmentation that is consistent with apoptosis (Figure 7H, arrow) and abnormal morphology that resulted in an inability to ensheath germ cells (Figure 7I, arrow). These phenotypes suggest that the loss of shi RNAi CySC clones is due to defects in cell survival and differentiation. CySC clones depleted for Crag were observed at similar rates to control clones at both 2 and 7 days ACI (Figure 7, A, J, J′, K, and K′), indicating that Crag is not required for CySC self-renewal or niche competition. However, Crag knockdown in CySC clones resulted in an unexpected phenotype, which has not been observed previously. In 90% of testes with Crag RNAi CySC clones, no labeled cells were found associated with differentiated germ cell cysts, suggesting that Crag-deficient cyst cells do not differentiate properly. In testes with control clones, differentiated-labeled cells moved distally over time (Figure 7J′′′, red arrows). Conversely, when Crag was knocked down in CySC clones, no labeled cells were observed distally in the testis, and instead Crag-deficient cyst cells accumulated apically and did not associate with germ cell cysts (Figure 7K′′′, green arrow). Thus, Crag appears to be dispensable for CySC function, but required for cyst cell differentiation; leading to clones that accumulate CySCs and early cyst cells.

## Discussion

Here we have generated a new resource for investigating the genetic basis of stem cell niche competition. Our collection of confirmed and candidate competition mutations demonstrates the breadth of cell functions that influence niche competition, and will allow for further study into many different facets of this process. We identified one hyper-competition mutation and seven additional candidate hyper-competition mutations in genes involved in a variety of functions, including mitochondrial function (*sicily*; Zhang *et al.* 2013), tumor suppression (*Rbf*; van den Heuvel and Dyson 2008), and protein ubiquitination (*bendless*; Muralidhar and Thomas 1993). The diversity of this list of candidates suggests that effects on many different cellular processes can lead to a hyper-competition phenotype. Yet the vast majority of mutations we have studied do not cause hyper-competition, suggesting that the ultimate cause(s) of hyper-competition are more constrained, and that the mechanism of hyper-competition may be similar in these diverse mutants.

A previously unstudied aspect of niche competition revealed by our screen is the involvement of vesicle trafficking genes. The *Sec16^A^*, *shi^A^*, *wus^A^*, and *Crag^A^* mutations that we have studied here represent a broad range of vesicle trafficking functions. Specifically, *Sec16* is a central regulator of exocytosis, whereas *wurst* and *shibire* function primarily during endocytosis (González-Gaitán 2003; Behr *et al.* 2007), and *Crag* is required in follicle cells for trafficking of basement membrane components to the basal surface (Denef *et al.* 2008). The *shi^A^* (K10X) and *wus^A^* (Q307X) alleles, which produced the most severe effects on DE-cad localization, contain nonsense mutations and are most likely to be amorphic alleles; while *Crag^A^* (C1372S) and *Sec16^A^* (P1926S) contain missense mutations and are more likely to be hypomorphic alleles. Our comparison of the relatively mild phenotypes caused by homozygosity for *Sec16^A^* to the more severe phenotypes caused by RNAi knockdown of *Sec16^A^* further support the conclusion that *Sec16^A^* is a hypomorphic allele. Interestingly, despite these differences, all four mutations caused strong hypo-competition phenotypes in the FSC lineage. This suggests that the process of niche competition is very sensitive to the function of these genes and is able to efficiently eliminate stem cell lineages with even only mild defects in vesicle trafficking.

Our findings suggest at least two possible reasons why impaired vesicle trafficking causes hypo-competition. First, DE-cad is known to be required for FSC maintenance (Song and Xie 2002), so the disruption of DE-cad localization to the membrane in vesicle trafficking mutants (Figure 5) could at least partially account for the hypo-competition phenotype. Second, vesicle trafficking is required for functional EGFR signaling in MDCK cells (Fogelgren *et al.* 2014), and we found decreased pERK levels in the vesicle trafficking mutant clones. Since EGFR signaling is also essential for FSC self-renewal (Castanieto *et al.* 2014), the reduction in EGFR signaling may be another factor that contributes to the hypo-competition phenotype. However, EGFR signaling is normally downregulated as cells exit the FSC niche region, so it is unclear whether the decreased pERK levels in these mutant clones are a cause of the hyper-competition phenotype or a consequence of reduced association with the niche (for example, because DE-cad levels on the membrane are low). In addition, it is also unclear why the loss of detectable pERK in these mutant clones is not associated with a cell polarity defect, as we observed previously in *EGFR* null clones (Castanieto *et al.* 2014). It may be that the vesicle trafficking alleles investigated in this study reduce the levels of EGFR signaling to a level that is below the limit of detection but sufficient to maintain cell polarity; or that the vesicle trafficking mutants impair the branch of EGFR signaling leading to ERK phosphorylation, but not the branch going through LKB1 and AMPK that is important for the maintenance of cell polarity in the FSC lineage. Alternately, it could be that the decrease in EGFR signaling is more gradual in the vesicle trafficking mutant clones than it is in *EGFR* mutant clones, so the vesicle trafficking mutant clones are able to grow normally for some time before EGFR signaling decreases to the point at which it is both undetectable by pERK staining and unable to promote FSC self-renewal or follicle cell polarity. Additional studies of these and other FSC niche competition mutants identified here will be important to clarify these issues.

In the testis, knockdown of *Sec16* also affected CySC niche competition rather than self-renewal or differentiation, whereas knockdown of *shi* likely impaired CySC self-renewal or survival. Interestingly, knockdown of *Crag* had no effect on CySC retention in the niche but caused an unusual differentiation defect in which mutant cells failed to move out of the niche region. These differences indicate that there are tissue-specific aspects to the process of self-renewal and niche competition, and provide additional evidence that the hypo-competition phenotypes we observed in the FSC niche are not due to a generic defect that would affect all cells equally. Overall, our studies demonstrate that mutations in multiple types of vesicle trafficking genes cause hypo-competition in both the ovary and testis. Vesicle trafficking is essential for a diverse array of cellular functions, and thus may function as a node, integrating outputs from these different cellular functions into a readout that influences the overall fitness of the cell for occupying the niche. Further investigation will make it possible to organize these and other mutations that cause niche competition phenotypes into common pathways and, ultimately, to understand whether and how stem cell niche competition promotes the maintenance of a healthy population of stem cells in each tissue throughout adulthood.

## Supplementary Material

Supplemental material is available online at www.genetics.org/lookup/suppl/doi:10.1534/genetics.117.201202/-/DC1.
