# Supplementary material for: Neutral Competition for Drosophila Follicle and Cyst Stem Cell Niches Requires Vesicle Trafficking Genes
Source: Genetics. 2017 May 16;206(3):1417–28. doi: 10.1534/genetics.117.201202 (PMC5500140; doi:10.1534/genetics.117.201202)
Supplement: Supplementary file 2 [file 1417FileS2.pdf]

Table S1

Gene	Mutation	Type	None	Single	Double	n=	Total Marked FSCs	χ^2 p-value
**Arp2**	W89stop	nonsense	100.0%	0.0%	0.0%	143	0.0%	5.9E-15
**CG11417**	R103stop	nonsense	100.0%	0.0%	0.0%	146	0.0%	3.1E-15
**CG8636**	K216stop	nonsense	100.0%	0.0%	0.0%	156	0.0%	3.5E-16
**Crag**	C1372S	missense	100.0%	0.0%	0.0%	153	0.0%	6.7E-16
**Cyp4d2**	K350X	nonsense	100.0%	0.0%	0.0%	128	0.0%	1.5E-13
**l(1)1Bi**	L285stop	nonsense	100.0%	0.0%	0.0%	62	0.0%	2.7E-07
**Nmd3**	V4516E	missense	100.0%	0.0%	0.0%	154	0.0%	5.4E-16
**Rhp**	M1K	missense	100.0%	0.0%	0.0%	93	0.0%	3.1E-10
**RpS5A**	Y80stop	nonsense	100.0%	0.0%	0.0%	70	0.0%	4.7E-08
**Tko**	Q6stop	nonsense	100.0%	0.0%	0.0%	115	0.0%	2.5E-12
**Upf1**	W120stop	nonsense	100.0%	0.0%	0.0%	98	0.0%	1.0E-10
**Wds**	G132N	missense	100.0%	0.0%	0.0%	82	0.0%	3.4E-09
**Zpr1**	C46S	missense	100.0%	0.0%	0.0%	26	0.0%	8.7E-04
**CDC45L**	Q540stop	nonsense	99.2%	0.8%	0.0%	125	0.8%	9.8E-13
**CG1703**	Q641stop	nonsense	99.0%	1.0%	0.0%	103	1.0%	1.2E-10
**Shi**	K28stop	nonsense	98.6%	1.4%	0.0%	69	1.4%	2.0E-07
**dsh**	(none found)	n/a	98.4%	1.6%	0.0%	123	1.6%	5.0E-12
**mRpL38**	Q88stop	nonsense	98.1%	1.9%	0.0%	157	1.9%	1.0E-14
**wap**	Y842stop	nonsense	97.9%	2.1%	0.0%	47	2.1%	2.6E-05
**CG7065**	K327stop	nonsense	96.3%	3.7%	0.0%	164	3.7%	6.9E-14
**CG34401**	W946stop	nonsense	96.2%	3.8%	0.0%	156	3.8%	3.8E-13
**CG11418**	Q47stop	nonsense	95.3%	4.7%	0.0%	172	4.7%	1.1E-13
**Phl**	L163R	missense	94.4%	5.6%	0.0%	36	5.6%	9.7E-04
**Wus**	Q307stop	nonsense	93.8%	6.3%	0.0%	32	6.3%	2.4E-03
**CG3704**	E300stop	nonsense	93.1%	6.9%	0.0%	102	6.9%	1.1E-07
**mus101**	Q525stop	nonsense	90.8%	9.2%	0.0%	174	9.2%	2.0E-10
**TH1**	R149stop	nonsense	89.2%	10.8%	0.0%	111	10.8%	1.9E-06
**CG3446**	E82V	missense	87.2%	12.8%	0.0%	86	12.8%	1.2E-04
**Inx2**	Q194stop	nonsense	88.0%	11.1%	0.9%	108	13.0%	1.9E-05
**flw**	K156stop	nonsense	88.3%	10.4%	1.3%	77	13.0%	3.0E-04
**Vps26**	K264stop	nonsense	86.5%	12.0%	1.5%	133	15.0%	1.7E-05
**Usf**	Y248N	missense	84.7%	13.6%	1.7%	59	16.9%	9.4E-03
**Kdn**	W370stop (PB isoform)	nonsense	87.5%	6.9%	5.6%	160	18.1%	6.4E-05
**CG15208**	L318F	missense	81.1%	17.9%	0.9%	106	19.8%	3.4E-03
**Cdk7**	E68K	missense	80.0%	20.0%	0.0%	50	20.0%	0.05
**Scu**	Q159stop	nonsense	79.2%	20.8%	0.0%	101	20.8%	7.4E-03
**dor**	Q208stop	nonsense	79.4%	19.6%	0.9%	107	21.5%	8.7E-03
**Aats-his**	D160V	missense	80.0%	17.2%	2.8%	180	22.8%	2.1E-03
**Egh**	D241N	missense	74.5%	23.6%	1.8%	110	27.3%	0.13
**COQ7**	Y90stop	nonsense	75.0%	22.5%	2.5%	40	27.5%	0.37

**Prosα4**	G36R	missense	75.0%	22.1%	2.9%	140	27.9%	0.11
**CG8184**	T1107I	missense	76.4%	18.5%	5.1%	178	28.7%	0.11
**CG1749**	K298stop	nonsense	74.5%	21.4%	4.1%	145	29.7%	0.22
**Stim**	W202stop	nonsense	73.3%	22.6%	4.1%	195	30.8%	0.26
**l(1)G0255**	V141M	missense	72.8%	23.5%	3.7%	136	30.9%	0.36
**CG3011**	M281R	missense	70.9%	26.3%	2.9%	175	32.0%	0.44
**DAAM**	N360V	missense	71.4%	24.8%	3.8%	105	32.4%	0.60
**br**	C665R	missense	74.1%	18.2%	7.7%	143	33.6%	0.73
**CG17776**	Y21stop	nonsense	71.3%	23.6%	5.1%	178	33.7%	0.72
**CG42237**	E361K	missense	70.7%	24.4%	4.9%	164	34.1%	0.81
**mew**	R165stop	nonsense	68.8%	28.1%	3.1%	160	34.4%	0.86
**Sp1**	Q8stop	nonsense	69.5%	26.3%	4.2%	118	34.7%	0.94
**Rap**	(not assessed)	n/a	69.6%	26.1%	4.3%	138	34.8%	0.94
**AP-1γ**	L133P	missense	68.8%	27.5%	3.7%	109	34.9%	0.96
**iso19A**	n/a	n/a	71.6%	21.6%	6.8%	148	35.1%	NA
**l(1)G0334**	G126E (PA isoform)	missense	70.5%	23.8%	5.7%	105	35.2%	0.98
**mst**	V216E	missense	68.0%	28.7%	3.3%	122	35.2%	0.98
**smox**	Q313stop	nonsense	70.1%	24.5%	5.4%	147	35.4%	0.96
**ewg**	Q80stop	nonsense	69.7%	23.6%	6.7%	89	37.1%	0.73
**Schlank**	S344P	missense	65.9%	29.5%	4.5%	132	38.6%	0.45
**Gtp-bp**	V439D	missense	66.7%	27.9%	5.4%	111	38.7%	0.48
**Tay**	R990stop	nonsense	68.0%	25.2%	6.8%	147	38.8%	0.41
**Pck**	P50L	missense	67.5%	23.4%	9.1%	77	41.6%	0.29
**CG13365**	V47D	missense	65.9%	26.2%	7.9%	214	42.1%	0.06
**dlg1**	splice donor	splice	67.5%	21.4%	11.1%	126	43.7%	0.08
**Sicily**	Q133stop	nonsense	63.4%	28.5%	8.1%	186	44.6%	0.02
**CG42749**	Q27X	missense	59.0%	30.8%	10.3%	117	51.3%	1.2E-03

Genes involved in vesicle trafficking are shaded in orange, the isogenized control is shaded in yellow, candidate hypercompetition alleles are shaded in green, and candidate hypocompetition alleles are shaded in blue.

Table S2

Gene	Mutation	Type	None	Single	Double	n=	Total Marked FSCs	χ^2 p-value
RpS5A	Y80stop	nonsense	100.0%	0.0%	0.0%	102	0.0%	7.1E-07
CG2918	Q619stop	nonsense	100.0%	0.0%	0.0%	84	0.0%	6.8E-06
CG4165	splice donor	splice	100.0%	0.0%	0.0%	55	0.0%	2.7E-04
Ck1α	L141M	missense	100.0%	0.0%	0.0%	80	0.0%	1.1E-05
eIF2B-ε	Y534stop	nonsense	100.0%	0.0%	0.0%	106	0.0%	4.3E-07
Mcm6	Q510stop	nonsense	100.0%	0.0%	0.0%	56	0.0%	2.4E-04
mRNA-cap	Y346stop	nonsense	100.0%	0.0%	0.0%	105	0.0%	4.9E-07
mxc	Q615stop	nonsense	100.0%	0.0%	0.0%	58	0.0%	1.8E-04
PpV	I117F	missense	100.0%	0.0%	0.0%	76	0.0%	1.9E-05
Psf3	Q141stop	nonsense	100.0%	0.0%	0.0%	78	0.0%	1.4E-05
Rbcn-3A	Q41stop	nonsense	100.0%	0.0%	0.0%	36	0.0%	3.2E-03
Rbcn-3B	R1294stop	nonsense	100.0%	0.0%	0.0%	79	0.0%	1.3E-05
RpII215	V785E	missense	100.0%	0.0%	0.0%	90	0.0%	3.2E-06
Sas10	Q177stop	nonsense	100.0%	0.0%	0.0%	94	0.0%	1.9E-06
SkpA	L22stop	nonsense	100.0%	0.0%	0.0%	94	0.0%	1.9E-06
vnd	splice acceptor	splice	100.0%	0.0%	0.0%	73	0.0%	2.7E-05
Ykt6	Q62R	missense	100.0%	0.0%	0.0%	96	0.0%	1.5E-06
sgg	P223L	missense	99.1%	0.9%	0.0%	114	0.9%	4.9E-07
CG3149	W535stop	nonsense	99.0%	1.0%	0.0%	96	1.0%	4.7E-06
CG1597	Y761N	missense	97.7%	2.3%	0.0%	44	2.3%	3.6E-03
temp	K14stop	nonsense	97.5%	2.5%	0.0%	80	2.5%	1.0E-04
por	R135stop	nonsense	97.4%	2.6%	0.0%	76	2.6%	1.7E-04
nej	Y1001N	missense	96.0%	4.0%	0.0%	25	4.0%	4.6E-02
Marf	A207V	missense	95.8%	4.2%	0.0%	120	4.2%	1.4E-05
mRpS25	splice donor	splice	95.3%	4.7%	0.0%	86	4.7%	3.6E-04
CG9650	splice donor	splice	94.2%	5.8%	0.0%	52	5.8%	9.6E-03
CG32649	E623K	missense	93.2%	6.8%	0.0%	59	6.8%	9.8E-03
β'COP	S570F	missense	90.7%	9.3%	0.0%	108	9.3%	3.6E-03
CG7358	L730stop	nonsense	90.4%	9.6%	0.0%	115	9.6%	3.4E-03
svr	S950T	missense	92.1%	6.3%	1.6%	63	9.5%	0.03
Smr	Q1655stop	nonsense	87.5%	12.5%	0.0%	72	12.5%	0.08
CG2025	W369stop	nonsense	88.0%	10.0%	2.0%	100	14.0%	0.09
dwg	C363S	missense	85.9%	14.1%	0.0%	64	14.1%	0.17
Myb	G111stop	nonsense	84.4%	15.6%	0.0%	109	15.6%	0.16
CG15896	Y121D	missense	83.2%	16.8%	0.0%	107	16.8%	0.27
pot	K551stop	nonsense	84.1%	14.0%	1.9%	107	17.8%	0.37
oc	Q469stop	nonsense	84.6%	12.3%	3.1%	65	18.5%	0.57
Fur2	splice donor	splice	81.6%	17.5%	0.9%	114	19.3%	0.59
β-Spec	W642stop	nonsense	82.2%	14.9%	3.0%	101	20.8%	0.87

iso19A	n/a	n/a	81.0%	16.5%	2.5%	79	21.5%	NA
CG14786	K313stop	nonsense	79.3%	19.0%	1.7%	116	22.4%	0.83
pI4KIIIα	W879stop	nonsense	78.8%	18.2%	3.0%	33	24.2%	0.72
mRpS30	splice donor	splice	77.3%	21.0%	1.7%	119	24.4%	0.48
CG17829	H253R	missense	79.5%	15.9%	4.5%	88	25.0%	0.46
Brms1	K188stop	nonsense	78.6%	17.5%	4.0%	126	25.4%	0.32
cac	W623stop	nonsense	77.0%	20.4%	2.7%	113	25.7%	0.31
para	N343I	missense	75.0%	22.6%	2.4%	84	27.4%	0.22
CG32795	A196V	missense	73.3%	25.3%	1.3%	75	28.0%	0.20
CG18624	stop57L	missense	76.0%	19.8%	4.1%	121	28.1%	0.10
comt	L257Q	missense	75.8%	18.9%	5.3%	95	29.5%	0.08
l(1)G0156	splice donor	splice	74.0%	22.0%	4.0%	100	30.0%	0.05
ATP7	R355stop	nonsense	73.8%	18.8%	7.5%	80	33.8%	0.01
Rbf	K458stop	nonsense	60.0%	32.7%	7.3%	110	47.3%	7.1E-10
ben	W129stop	nonsense	52.9%	29.0%	18.1%	138	65.2%	1.1E-31

Genes involved in vesicle trafficking are shaded in orange, the isogenized control is shaded in yellow, candidate hypercompetition alleles are shaded in green, and candidate hypocompetition alleles are shaded in blue.
